# Incremental diagnostic utility of delayed enhancement CMR tissue characterization for detection of catheter associated right atrial thrombus

**DOI:** 10.1186/1532-429X-17-S1-P327

**Published:** 2015-02-03

**Authors:** Andrew Plodkowski, Nina Kukar, Dipti Gupta, Yulia Lakhman, Jiwon Kim, Jennifer Liu, Nancy Roistacher, Stephen Solomon, Richard Steingart, Jonathan W Weinsaft

**Affiliations:** 1 Memorial Sloan Kettering Cancer Center, New York, NY, USA; 2 Weill Cornell Medical College, New York, NY, USA

## Background

Right atrial thrombus (RT) provides a rationale for anticoagulation and substrate for embolic events. CMR is well validated for thrombus detection, but has yet to be used to assess prevalence and predictors of RA thrombus among at risk cohorts.

## Methods

The population comprised consecutive patients with central venous catheters undergoing CMR at Memorial Sloan Kettering Cancer Center (NY, NY). Delayed enhancement CMR (inversion recovery GRE) was used to identify RT; defined as a right atrial (RA) mass with avascular tissue characteristics (non-enhancing) on "long TI" (600msec) DE-CMR. Cine-CMR (SSFP) was used to quantify cardiac structure and function, including RA and RV function and chamber size. Clinical indices were categorized based on medical record review. Echo (if performed within 14 days of CMR) was retrieved from image archives and independently read for RT. Clinical records were queried for documented pulmonary embolus (PE) within 60 days of CMR.

## Results

50 cancer patients (50±17yo, 64% female) with RA catheters were studied. CMR was performed for evaluation of a suspected RA mass (36%) or unrelated clinical indications (64%). RT was present in 22% (n=11); all had RT avascularity confirmed by dedicated "long TI" DE-CMR. Among affected patients, 63% had a solitary RT (36% multiple). Patients with RT had similar right-sided structure and function vs. those without RT based on RA end-diastolic area (10.2±3.5 vs. 10.2±2.1 cm^2^/m^2^, p= 0.94), RA end-systolic area (6.9±3.7 vs. 6.6±1.9 cm^2^/m^2^, p=0.76), RV end-diastolic volume (73±21 vs. 67±16 ml/m^2^, p= 0.27), and RVEF (57±8 vs. 59±9%, p= 0.40). Cancer diagnosis (73 vs. 85% solid tumor, p=0.39), catheter depth (2.3±2.2 vs. 2.1±1.8cm from RA/SVC junction, p=0.74), age and gender (both p=NS) were similar between groups. Transthoracic echo, attained 4.1±3.8 days from CMR in 50% of the population, demonstrated high sensitivity (89%) but moderate specificity (75%) in relation to DE-CMR. Cine-CMR yielded similar sensitivity (82%) but improved specificity (97%) vs. the reference standard of DE-CMR (Table). 27% of patients (3/11) with RT on DE-CMR had PE; all occurred prior to DE-CMR (average of 14 days before). Conversely, no PEs occurred among patients without RT. Clinical embolic events were independent of RT size (3.0±2.6cm^2^ vs. 2.6±1.3cm^2^, p=0.75).

## Conclusions

Catheter associated RT occurs independently of right-sided structure or function, and is associated with clinical embolic events. Morphologic imaging by cine-CMR and echo provide limited diagnostic utility for RT as established by DE-CMR tissue characterization.

## Funding

None.

**Table 1:** Diagnostic performance of anatomic imaging for right atrial thrombus by DE-CMR tissue characterization.

	Sensitivity	Specificity	Accuracy	PPV	NPV
Cine-CMR	82% (9/11)	97% (38/39)	94% (47/50)	90% (9/10)	95% (38/40)

Transthoracic Echo*	89% (8/9)	75% (12/16)	80% (20/25)	67% (8/12)	92% (12/13)

*obtained in 50% of study population (n=25)

